# Case Report: A new *UBA2* variant in a Chinese family with aplasia cutis congenita

**DOI:** 10.3389/fmed.2026.1819096

**Published:** 2026-05-08

**Authors:** Yang He, Shengcai Zhu, Quan Wei, Fan Ye, Xiaoliang Ouyang, Liang Wu, Chunming Li

**Affiliations:** 1 Department of Dermatology, The Second Affiliated Hospital, Jiangxi Medical College, Nanchang University, Nanchang, Jiangxi, China; 2 Department of Plastic Surgery, The Second Affiliated Hospital, Jiangxi Medical College, Nanchang University, Nanchang, Jiangxi, China

**Keywords:** aplasia cutis congenita, Chinese, *UBA2*, variant, whole-exome sequencing

## Abstract

Aplasia cutis congenita (ACC) is a congenital disorder characterized by localized absence of skin and subcutaneous tissue, which can occur either in isolation or as a component of syndromic conditions. Here, we describe a Chinese family with isolated ACC involving a 6-month-old male proband and his mother, both harboring a novel heterozygous missense variant in the *UBA2* gene (NM_005499.3):c.365G > A, p.Arg122Gln. This variant, located in the functionally critical UBA-e1-thiolCys domain, was identified by whole-exome sequencing and validated via Sanger sequencing in both affected individuals. Our findings expand the genotypic spectrum of *UBA2*-related disorders, highlighting the association between *UBA2* variants and non-syndromic inherited ACC.

## Introduction

Aplasia Cutis Congenita (ACC) is a rare congenital anomaly characterized by a localized absence of skin, predominantly affecting the scalp. In some instances, the defect may extend into deeper structures, involving the dura mater or osseous structures. The first case of ACC was described in 1767 by Cordon. Globally, the estimated incidence of ACC is approximately 3 in 10,000 live births (1). Histologically, ACC typically exhibits a complete absence of epidermis. Clinically, ACC must be distinguished from intrauterine herpetic infections or birth trauma. While the majority of patients with ACC do not present with other congenital abnormalities, it can occur as part of rare syndromes that encompass a wide spectrum of anomalies. Syndromic congenital skin defects associated with ACC include Johanson–Blizzard syndrome (OMIM #243800), Adams–Oliver syndrome (AOS) (OMIM #100300), and scalp-ear-nipple syndrome (OMIM #181270), among others (2). In this report, we describe a Chinese family in which both the mother and son are affected by ACC and have been found to carry a heterozygous variant in the *UBA2* gene: c.365G > A, p.Arg122Gln.

## Cases presentation and analysis of the variant

**FIGURE 1:**
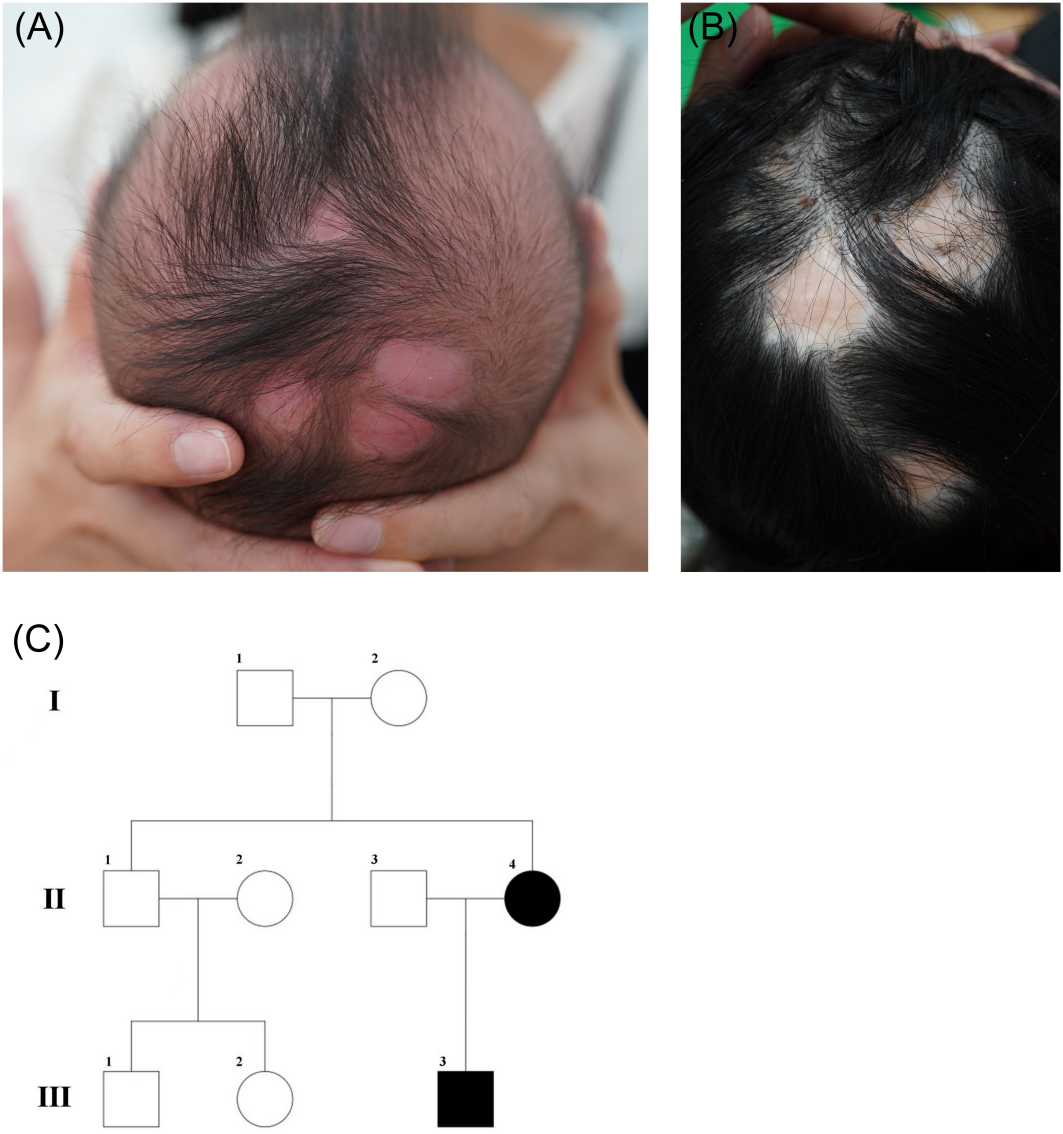
**(A)** Clinical image of the proband. **(B)** Clinical image of the probands’ mother. **(C)** Pedigree of the affected family with Aplasia cutis congenita (ACC). The square represents males and while the circle represents females. The affected members with ACC are depicted in black.

The proband, a 6-month-old infant, presented to our hospital with a skin defect localized to the scalp since birth. His birth length, weight, and head circumference were within normal limits, and he did not sustain any birth injuries or exhibit additional congenital anomalies. The prenatal course was unremarkable, with no reported history of teratogenic exposures. Physical examination revealed four well-circumscribed localized lesions, each ranging from 0.8 to 1.5 cm in diameter, situated on the crown of the proband’s head (Figure 1A). The proband’s mother also exhibited isolated aplasia cutis on the scalp at birth and was otherwise in good health (Figure 1B). No other family members (including the proband’s father) reported similar symptoms. The family pedigree is illustrated in Figure 1C.

**FIGURE 2:**
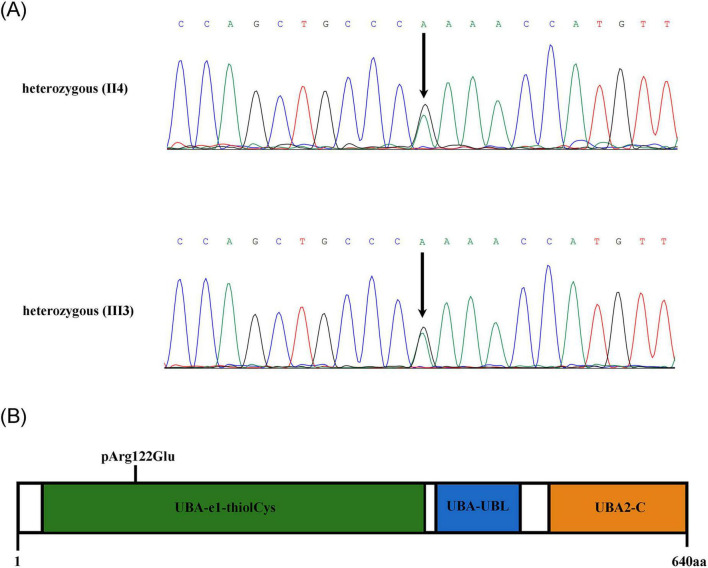
**(A)** DNA sequence analysis identified a novel variant (c.365G > A) in *UBA2* in both the proband and his mother. **(B)** Schematic representation of the UBA2 protein structure. UBA2 has three domains: UBA-e1-thiolCys, UBA-UBL, and UBA2-C.

Following ethics approval and informed consent, genomic DNA was extracted from the patient’s peripheral blood for whole-exome sequencing. This analysis revealed a missense variant (NM_005499.3:c.365G > A, p.Arg122Gln) in the *UBA2* gene, which has not been reported in the literature or in any public databases, including the 1000 Genomes Project, ESP6500, ExAC, gnomAD, ClinVar, and HGMD. Therefore, it is considered a novel variant. To confirm the presence of the variant, Sanger sequencing was performed on both the proband and his mother, identifying the *UBA2* variant c.365G > A as a heterozygous variant in both individuals (Figure 2A). This variant is located within the UBA-e1-thiolCys domain (Figure 2B).

**FIGURE 3:**
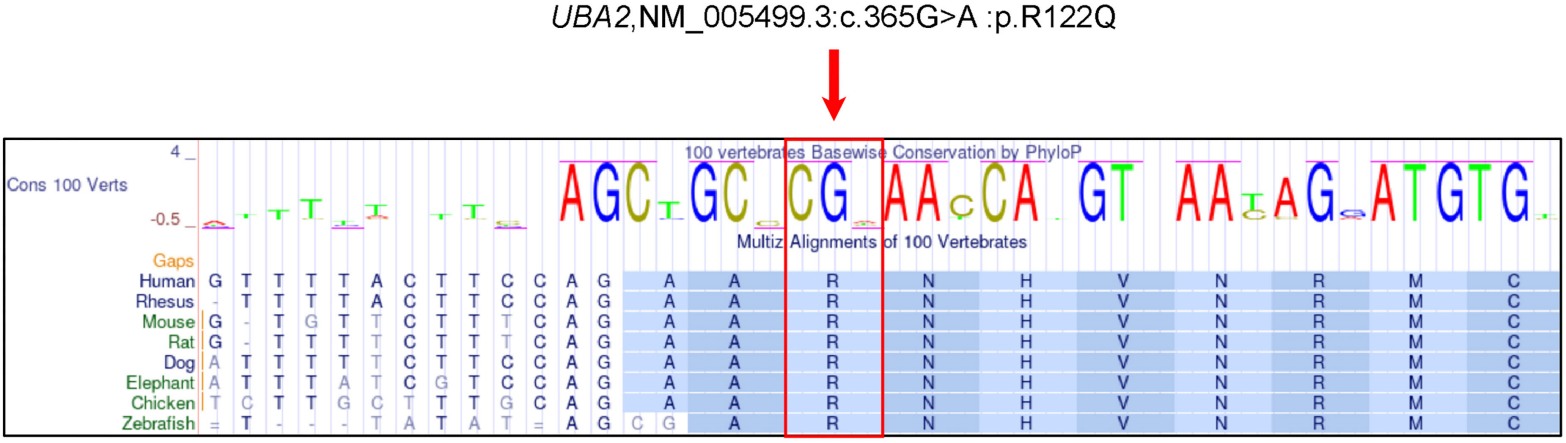
Conservation of amino acid sequences in the corresponding variant of *UBA2* between species. The red arrow represents the amino acid at the mutated site.

The UCSC Genome Browser^1^ was utilized to investigate the evolutionary conservation of the amino acids involved in the variant. An alignment of multiple sequences of homologous proteins from humans and various other vertebrates revealed that the *UBA2* c.365G > A variant occurred at highly conserved sites (Figure 3).

**FIGURE 4:**
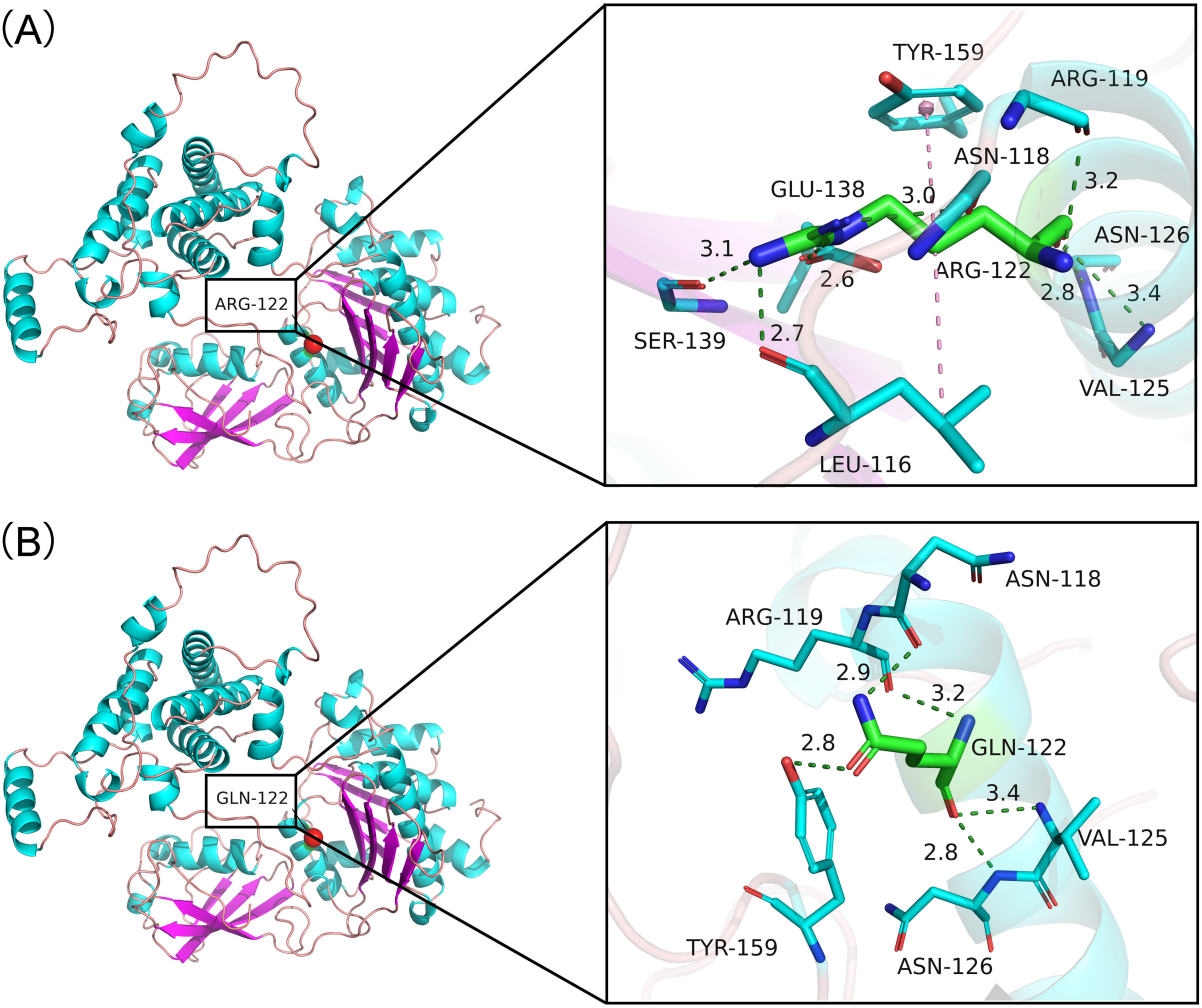
Three-dimensional structure of wild-type proteins and the novel mutant proteins. **(A)** Wild-type protein: its side chains form hydrogen bonds with Ser139, Asn118, Leu116, and Glu138, while the main chains form hydrogen bonds with Val125, Asn126, and Arg119 of the protein. **(B)** Mutant protein: the side chains of the mutant form hydrogen bonds with Tyr159 and Asn118, while the main chains form hydrogen bonds with Asn126, Val125 and Arg119 of the protein.

To further explore the molecular structures of the wild type (WT) and mutant protein, we constructed three-dimensional structural models using the I-TASSER server^2^. As illustrated in Figure 4, the WT protein contains a polar arginine residue at position 122. Its side chains form hydrogen bonds with Ser139, Asn118, Leu116, and Glu138, while the main chains form hydrogen bonds with Val125, Asn126, and Arg119 of the protein. In contrast, the p. Arg122Gln variant substitutes Arg122 with Gln. The side chains in the mutant form hydrogen bonds with Tyr159 and Asn118, while the main chains form hydrogen bonds with Asn126, Val125, and Arg119 of the protein.

To predict the likelihood of pathogenicity, four online tools (PROVEAN, PolyPhen-2, SIFT, Mutation Taster)^3^ ^4^ ^5^ ^6^ were employed. The novel UBA2 c.365G > A variant was classified as “disease causing” by MutationTaster (score = 0.999), “probably damaging” by PolyPhen2 (score = 1.000), “Damaging” (score = 0.000) by SIFT, and “deleterious” (score = −3.52) by PROVEAN. These results support the pathogenicity of the variant carried by the proband and his mother.

## Discussion

*UBA2* is a gene located in the chromosomal region 19q13.11, with a size of 42.9 kb and consisting of 17 exons. This gene encodes Ubiquitin-Like Modifier Activating Enzyme 2 (UBA2), which forms a heterodimeric complex with SAE1 (SUMO-Activating Enzyme 1). Together, these enzymes function as E1 enzymes in the SUMOylation cascade. The activated UBA2-SAE1 complex transfers Small Ubiquitin-like Modifier (SUMO) to the E2 enzyme in the SUMOylation pathway, thereby regulating various cellular processes, including proliferation, migration, stress response, and tumorigenesis. The acute knockdown of *UBA2* in xenograft tumors using conditional short hairpin RNA (shRNAs) results in significant growth arrest, defects in cell proliferation, and increased apoptosis (3). An *in situ* hybridization study conducted on mouse embryos revealed substantial expression of *UBA2* at multiple sites of morphogenetic activity, including the neural folds, branchial arches, and limb buds, suggesting that *UBA2* is essential for normal cellular function and development (4). In *Caenorhabditis elegans*, *UBA2* is recognized as a critical component of the SUMOylation pathway; its ablation leads to embryonic lethality (5). Additionally, *UBA2* is expressed in the eyes, brains, and pectoral fins of zebrafish, and *UBA2*-null fish exhibit impaired growth, microcephaly, microphthalmia, mandibular hypoplasia, and abnormal fins (6).

The present study identified a novel pathogenic variant, *UBA2* c.365G > A (p.Arg122Gln), in a Chinese family. This variant results in the substitution of arginine with glutamine at codon 122. A previous study also reported that *UBA2* variants lead to amino acid changes at this position, specifically *UBA2* c.364C > T (p.Arg122*) in an American family and c.364C > G (p.Arg122Gly) in a Canadian family (6). Furthermore, the *UBA2* c.365G > A variant is located within the UBA-e1-thiolCys domain, which features putative active sites for binding ATP, substrates, and zinc. A previous study revealed that the p.Arg122 substitution is predicted to result in the loss of interaction with ATP (5). Additionally, the protein structure prediction model suggests that the p.Arg122Gln variant replaces Arg122 with Gln, leading to hydrogen bond interactions between the side chains of the mutant form and Tyr159 and Asn118, while the main chains form hydrogen bonds with Asn126, Val125, and Arg119 of the protein. We speculate that these alterations in hydrogen bond interactions induce conformational changes and functional impairments in the protein, contributing to the onset of ACC.

Previous studies have hypothesized that *UBA2* plays a role in scalp defects associated with the 19q13.11 deletion syndrome and has been proposed as a novel candidate for Mendelian scalp defects and other anomalies. These anomalies include ACC, ectrodactyly, hand/foot malformations, neurodevelopmental disorders, hypotonia, and renal and genital abnormalities (6, 7). Recently, several studies have identified *UBA2* variants associated with Aplasia Cutis Congenita with Ectrodactyly syndrome (ACCES) (8, 9). Moreover, *UBA2*-related syndromic congenital skin defects, such as split-hand disorders, may exhibit incomplete penetrance (10). Therefore, although our proband has not exhibited any congenital abnormalities at present, long-term follow-up and observation are still necessary. Notably, the proband’s mother exhibited isolated aplasia cutis at birth and was otherwise in good health. *UBA2* variants associated with non-syndromic ACC are extremely rare. Wang et al. reported an inherited *UBA2* frameshift variant (c.327delT, p.Phe109Leufs*3) in a young boy and his mother. The mother had isolated cutis aplasia but was otherwise healthy (10). Our patients’ phenotypes and molecular findings indicate that *UBA2* should be included in the genetic differential diagnosis of aplasia cutis congenita (ACC), highlighting the importance of *UBA2* in the diagnostic evaluation of this condition.

This study has several limitations. Firstly, the blood sample from the proband’s father was not available for genetic analysis. Secondly, CT and MRI of the deeper structures were not performed due to the parents’ disagreement. Therefore, the involvement of the dura or bone cannot be ruled out.

## Conclusion

In summary, we report a Chinese family with ACC. We have identified a novel variant within this family, which enhances our understanding of the genotypic and phenotypic spectrum associated with *UBA2*-related disorders.

## Data Availability

The datasets presented in this study can be found in online repositories. The names of the repository/repositories and accession number(s) can be found below: https://www.ncbi.nlm.nih.gov/, PX910297.

## Footnotes

1
https://genome.ucsc.edu

2
http://zhanglab.ccmb.med.umich.edu/I-TASSER

3
http://provean.jcvi.org/index.php

4
http://genetics.bwh.harvard.edu/pph2

5
http://sift-dna.org/

6
http://www.mutationtaster.org
